# Management of postpartum pulmonary embolism combined with retained placenta accreta: A case report

**DOI:** 10.1097/MD.0000000000017219

**Published:** 2019-09-20

**Authors:** An Tong, Fumin Zhao, Ping Liu, Xia Zhao, Xiaorong Qi

**Affiliations:** a Department of Gynecology and Obstetrics, Development and Related Diseases of Women and Children Key Laboratory of Sichuan Province, Key Laboratory of Birth Defects and Related Diseases of Women and Children, Ministry of Education; b Department of Radiology, West China Second Hospital, Sichuan University, Chengdu, P.R. China.

**Keywords:** anticoagulant therapy, hysteroscopic resection, pulmonary embolism, retained placenta accreta

## Abstract

**Rationale:**

Retained placenta accreta is an increasing obstetric problem in recent years, and pulmonary embolism (PE) during pregnancy and the postpartum period is a vital condition, but lack of standard therapy guidelines. This report describes a case of postpartum PE combined with retained placenta accreta.

**Patient concerns:**

A 27-year-old woman presenting with fever and dyspnea after delivery was admitted to our hospital with retained placenta accreta.

**Diagnoses:**

The patient was diagnosed with the infection, postpartum PE, and residual placenta.

**Interventions:**

The antibiotics and low molecular weight heparin were initially started to cure the infection and control PE. Mifepristone was then used to promote the necrosis of residual placenta while long-term use of warfarin was served as continuous anticoagulant therapy. Hysteroscopic resection of retained placenta was not performed until thrombi had been almost disappeared after more than 2 months of anticoagulation therapy.

**Outcomes:**

The patient's menstruation returned to normal within several weeks after hysteroscopic resection and she completely recovered from PE after 3 months of anticoagulant therapy.

**Lessons:**

Treatment of retained placenta accreta can be postponed when encountering complicated cases, such as postpartum PE. PE in perinatal stage can be managed referring to nonmaternal PE.

## 1 Introduction

Retained placenta accreta is a rare condition and defined as a morbid adhesion of placenta to the uterine wall that can not be separated naturally after delivery.^[1]^ It has been an increasing challenge due to the rise in the cesarean section rate and artificial abortion rates,^[2]^ especially in China. Retained placenta accreta can lead to hemorrhage, infection, or surgical complications, even maternal deaths.^[1]^ However, the optimal treatment remains controversial regarding whether to remove the placenta in time by surgery or to leave the placenta with conservative treatment.^[3]^

As the leading cause of maternal mortality in the developed world,^[4]^ Pulmonary embolism (PE) has an increasing incidence during pregnancy and most often happens during the postpartum period. However, there are few reports of postpartum PE accompanied with retained placenta accreta. Here, we present a successfully treated case.

## 2 Case report

A 27-year-old woman (gravida 5, para 1) presenting with repeated fever and dyspnea was referred from a local hospital to our hospital for retained placenta. The patient had induced labor at 18 + 5 weeks gestation and subsequent curettage for adherent placenta 3 years ago. Ten days before the referral, she had a vaginal delivery at 40 weeks gestation and only partial placenta was manually removed due to placenta adhesion. Curettage was performed and postoperative ultrasound showed intrauterine scattered echogenic foci. In this situation, oxytocin was released continuously. Despite all this, the uterine contraction was good with little vaginal bleeding.

The patient developed a fever with a maximum temperature of 40.5°C 3 days later. Gram-negative bacilli were isolated from intrauterine secretion and ultrasound revealed placenta tissue measuring 7.3 × 3.6 × 6.0 cm^3^ without a clear boundary with myometrium. She was treated with meropenem 0.5 g, 8 hourly for 3 days, but the fever was still out of control. Thus, she was transferred to a superior hospital. Magnetic resonance imaging (MRI) revealed intrauterine placenta residue and partial penetration. Tienam 0.5 g, 6 hourly was used for 3 days and her body temperature raised to 42°C. Moreover, dyspnea was developed and *Staphylococcus epidermidis* was found in blood culture. Vancomycin 1 g, 12 hourly was added to the anti-infective therapy and continuous low flow oxygen was given. Laboratory result on serum procalcitonin was 1.49 ng/L and white blood cell count was 10.28 × 10^9^/L and hemoglobin 84 g/L.

**Figure 1:**
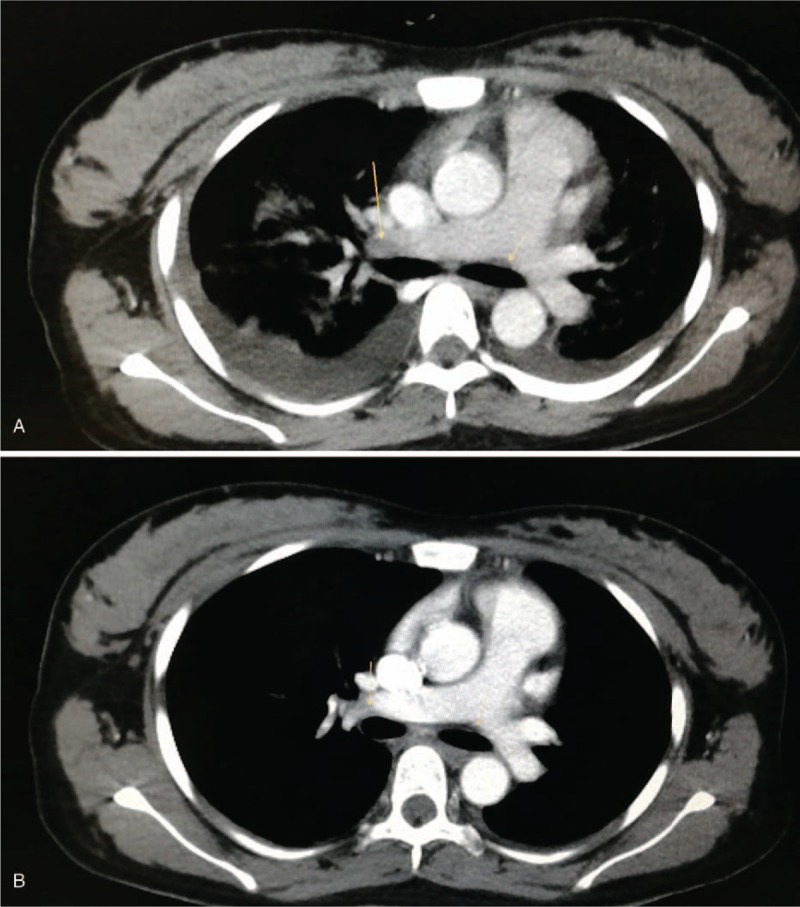
Contrast-enhanced CT scan revealed hypodense filling defects in pulmonary arteries (A) and re-examination showed reduction of the hypodense filling defects (B) after anticoagulant therapy. CT = computed tomography.

In consideration of deterioration of symptoms, the patient was referred to the emergency department in our hospital. Computed tomography (CT) of the chest revealed pneumonia and pleural effusion. After consultation of the infectious diseases department and the respiratory department, the patient continued with current treatment and was admitted to the intensive care unit (ICU) for further treatment. In the ICU, the patient had an intermittent fever with body temperature fluctuated from 36.5°C to 39.1°C accompanied with cough and dyspnea. Surplus pulse O_2_ (SpO_2_) maintained at 92% to 96% with oxygen-induced by nasal cannula at 4 L/min. The coagulation function test performed immediately after entering the ICU showed antithrombin III was 56%, fibrinogen degradation product was 8.6 μg/mL and D-dimer was 2.31 mg/L. Other coagulation parameters such as fibrinogen, prothrombin time and activated partial thromboplastin time were within normal ranges. The blood gas analysis revealed pH was 7.56, PCO2 was 33.75 mmHg, base excess was 7.6 mmol/L and HCO3 was 30.9 mmol/L and suggested a combination of metabolic acidosis and respiratory alkalosis. Hypodense filling defects in the bifurcation of pulmonary artery trunk and right main pulmonary artery, respectively (Fig. 1A) and pulmonary interstitial edema were found by contrast-enhanced CT. Additionally, pneumonia and pleural effusion were aggravated compared to the old CT image. According to the clinical manifestations, CT image and results of laboratory tests, a diagnosis of PE was established.

Given that the severe infection and PE, curettage for placenta residue should be delayed and treatment should be focused on controlling the infection and PE. Therefore, antibiotics continued on current protocol, diuretic and albumin were given and 0.4 mL Clexane was injected subcutaneously 12 hourly. Warfarin (2.5 mg per day) was added to anticoagulant therapy after consultation the next day. The level of the international normalized ratio (INR) was monitored daily. The patient's condition improved. On the fifth day of anticoagulant therapy, warfarin was adjusted to 3.75 mg per day due to INR was 1.13 and no hemorrhagic tendency.

On the seventh day in ICU, contrast-enhanced CT revealed disappearance of the hypodense filling defect in the pulmonary artery trunk and reduction in the right main pulmonary artery (Fig. 1B), alleviated edema and pneumonia and reduced pleural effusion. On the eighth day when the body temperature was within normal ranges for 3 consecutive days, antibiotic was changed to 4.5 g Tazocin, 8 hourly. The SpO_2_ remained good without oxygen intake. Clexane was stopped and warfarin was used alone. The patient was discharged from the ICU to general wards for continued therapy the next day.

On the twelveth day in our hospital, as the patient's condition stabilized, intravenous anti-infective therapy was stopped and oral administration of Cefdinir capsules was prescribed. Mifepristone 10 mg per day orally was started to promote necrosis of residual placenta. Warfarin was continued with 3.75 mg per day. The patient was discharged the next day.

After 1 month of outpatient treatment, rivaroxaban tablets were prescribed instead of warfarin for continued anticoagulant therapy. Another month later, the patient underwent hysteroscopic resection of retained placenta. Postoperative ultrasound demonstrated no abnormalities and her menstruation returned to normal within several weeks. After 3 months of anticoagulant therapy, pulmonary perfusion scan showed normal findings which suggested completed recovery of PE.

## 3 Discussion

Retained placenta accreta is an unclear disorder which may be the result of deficient decidua, excessive invasion of trophoblasts, and variations in maternal vascularity separately or in combination.^[5]^ A number of postulated risk factors for morbid adherence of placenta are dilatation and curettage, placenta previa, previous cesarean section, and history of uterine surgery or injury.^[6,7]^ In our case, the history of uterine manipulation was strong evidence for her placenta residue. Ultrasound, especially color Doppler ultrasonography, was a common method for detecting postoperative intrauterine condition for placenta accreta.^[8]^ Further examinations by 3-dimensional computed tomographic angiography can provide more precise information of vascularity in the retained placenta.^[9]^ MRI can provide a visual attachment site and depth of the placenta and help to design therapy project.^[10,11]^ Retained placenta accreta can lead to postpartum hemorrhage, which is the most common and severe complication for postoperative maternal morbidity, and intrauterine infection.^[12]^ It is reported that fever is a common complication of placenta accreta as a secondary symptom to sepsis or endomyometritis in most cases,^[1]^ which was also seen in our case. However, fever can also be the result of tissue necrosis without any inflammatory response.^[1]^ If the residual placenta is not removed in time, it may cause intrauterine adhesion or amenorrhea which will lead to infertility problems.^[13]^ In our case, although the patient came with PE and severe infection and the treatment was delayed, she recovered well without any complications.

Treatment for retained placenta accreta can vary from person to person in the absence of standard treatment guidelines. Medicine, such as methotrexate and mifepristone, can promote the necrosis and resorption of the placenta and is often used before curettage as the first option for retained placenta accreta.^[1]^ Mifepristone is preferred to methotrexate because it is more effective with fewer side effects.^[14]^ Transcatheter uterine arterial embolization is usually used in complicated cases, particularly those with significant bleeding tendency.^[15]^ Hysteroscopic resection as the most effective way is suitable for patients who can not completely remove the intrauterine residues after curettage or long-term retained placenta,^[16]^ as it is in our case. Other conservative treatments including uterotonic drugs and prophylactic antibiotic therapy are also involved as a combined therapy.^[17]^ One study showed that the success rate of conservative treatment was 78.5% (131/167) and the rest had hysterectomy.^[17]^

PE is a disease usually caused by emboli that block the pulmonary circulation, such as thrombus, gas, adipocytes, amniotic fluid, tumor, bacteria, or trophoblastic tissue.^[16]^ Pregnant women have a higher risk of PE, because venous stasis, vascular damage, and hypercoagulopathy, as the elements of Virchow's triad, are all existent during pregnancy and the postpartum period and contribute to thrombosis.^[18]^ It is important to detect and treat PE early. The diagnostic criteria of maternal PE are same as nonmaternal PE.^[19]^ The diagnosis is based on the clinical symptoms, such as respiratory arrest, hypotension, and loss of consciousness together with imaging findings such as diminished lung perfusion assessed by ventilation-perfusion scintigraphy or thrombi detected by CT pulmonary angiography or ultrasonography.^[19]^ In our case, the patient had dyspnea and thrombi found by contrast-enhanced CT, thus leading to the diagnosis of PE.

Treatment of PE in perinatal stage is largely based on extrapolation from non-pregnant cohorts or expert consensus as lack of randomized trials in pregnant. The anticoagulation with low molecular weight heparin is often the optimal treatment for pulmonary thromboembolism due to no teratogenic effect as unfractionated heparin does not cross the placenta. It has negligible risk for heparin-induced thrombocytopenia and osteoporosis, but good bioavailability and a predictive dose-response.^[20]^ Considering fetal exposure, warfarin can be used for nursing mothers but is not recommended for the patient in pregnancy.^[21]^ Our case is a postpartum PE which is suitable for using warfarin. It is reported that PE treated with thrombolytics has a favorable maternal outcome, but a substantial risk of maternal major bleeding is also observed.^[22]^ Surgical embolectomy is reserved for management of massive PE or when conservative treatment has little effect.^[22,23]^ Studies suggest that extracorporeal membrane oxygenation can be a new treatment approach for high-risk patients.^[22]^

## 4 Conclusion

In conclusion, retained placenta accreta should be treated in time, except complicated cases like ours which is accompanied with PE. Treatment of retained placenta accreta should be based on the actual situation, especially the vascular condition and depth of placental implantation, as well as complications. It can be referred to non-maternal PE for PE in perinatal stage as there is no standard therapeutic principle.

## Author contributions

**Conceptualization:** An Tong, Xiaorong Qi.

**Investigation:** An Tong.

**Resources:** Fumin Zhao.

**Supervision:** Xia Zhao.

**Writing – original draft:** An Tong.

**Writing – review & editing:** Ping Liu, Xia Zhao, Xiaorong Qi.

An Tong orcid: 0000-0002-5647-4884.
